# Mayo Genetic Risk Models for Newly Diagnosed Acute Myeloid Leukemia Treated With Venetoclax + Hypomethylating Agent

**DOI:** 10.1002/ajh.27564

**Published:** 2024-12-13

**Authors:** Naseema Gangat, Azeem Elbeih, Nour Ghosoun, Kristen McCullough, Fnu Aperna, Isla M. Johnson, Maymona Abdelmagid, Aref Al‐Kali, Hassan B. Alkhateeb, Kebede H. Begna, Michelle Elliott, Abhishek Mangaonkar, Aasiya Matin, Antoine N. Saliba, Mehrdad Hefazi Torghabeh, Mark R. Litzow, William Hogan, Mithun Shah, Mrinal M. Patnaik, Animesh Pardanani, Talha Badar, Hemant Murthy, James Foran, Jeanne Palmer, Lisa Sproat, Nandita Khera, Cecilia Arana Yi, Samuel Yates, Abigail Sneider, Emily Dworkin, Anand A. Patel, Alexandre Bazinet, Jayastu Senapati, Alex Bataller, Courtney DiNardo, Tapan Kadia, Ayalew Tefferi

**Affiliations:** ^1^ Division of Hematology Mayo Clinic Rochester Minnesota USA; ^2^ Division of Hematology Mayo Clinic Jacksonville Florida USA; ^3^ Division of Hematology Mayo Clinic Scottsdale Arizona USA; ^4^ Section of Hematology‐Oncology, Department of Medicine University of Chicago Chicago Illinois USA; ^5^ Department of Medicine University of Chicago Chicago Illinois USA; ^6^ Department of Pharmacy University of Chicago Chicago Illinois USA; ^7^ Department of Leukemia MD Anderson Cancer Center Houston Texas USA

**Keywords:** karyotype, mutations, remission, survival, venetoclax

## Abstract

Patients with newly diagnosed acute myeloid leukemia (ND‐AML) derive variable survival benefit from venetoclax + hypomethylating agent (Ven‐HMA) therapy. The primary objective in the current study was to develop genetic risk models that are predictive of survival and are applicable at the time of diagnosis and after establishing treatment response. Among 400 ND‐AML patients treated with Ven‐HMA at the Mayo Clinic, 247 (62%) achieved complete remission with (CR) or without (CRi) count recovery. Multivariable analysis–derived hazard ratios (HR), including 1.8 for European LeukemiaNet (ELN) adverse karyotype, 4.7 for *KMT2Ar*, 1.7 for *TP53* ^MUT^, 2.6 for *KRAS* ^MUT^, and 2.1 for *IDH2* ^WT^ were applied to develop an HR‐weighted risk model: low, intermediate, and high; respective median survival censored for allogeneic stem cell transplant (ASCT) (3‐year survival) were “not reached” (67%), 19.1 (33%), and 7.1 months (0%). In patients achieving CR/CRi, adverse karyotype, *KMT2Ar*, *KRAS* ^MUT^, *IDH2* ^WT^ predicted inferior survival, allowing for a complementary response‐stratified risk model. The model was externally validated and was shown to be superior to the ELN 2024 risk model (AIC 179 vs. 195 and AUC 0.77 vs. 0.69). Survival was inferior with failure to achieve CR/CRi or not receiving ASCT; 3‐year survival for high‐risk with or without ASCT was 42% versus 0% (*p* < 0.01); intermediate 72% versus 43% (*p* = 0.06); and low‐risk 88% versus 78% (*p* = 0.53). The Mayo genetic risk models offer pre‐treatment and response‐based prognostic tools for ND‐AML treated with Ven‐HMA. The current study underscores the prognostically indispensable role of achieving CR/CRi and ASCT.

## Graphical abstract

The Mayo genetic risk models offer pre‐treatment and response‐based prognostic tools for newly diagnosed AML treated with Ven‐HMA.

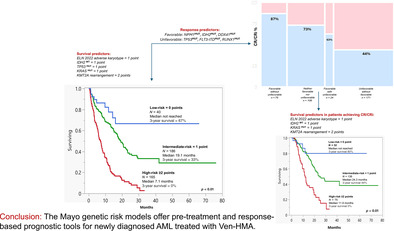

## 1 Introduction

Venetoclax + hypomethylating agent (Ven‐HMA) combination therapy has shown favorable response rates and overall survival (OS) in unfit patients with newly diagnosed acute myeloid leukemia (ND‐AML) [1, 2, 3]. However, there remains substantial heterogeneity in survival outcomes [4, 5]. Achievement of complete remission with (CR) or without (CRi) count recovery has long been recognized to have independent association with improved survival in AML while refractory/relapsed disease is associated with significantly shorter survival. In the seminal phase 3 VIALE‐A study [6], Ven‐azacitidine was compared to azacitidine‐placebo, in ND‐AML, and resulted in CR/CRi of 66% with a median OS of 14.7 months. The study also showed significantly longer OS in patients without versus with measurable residual disease (median 34.2 vs. 18.7 months) [6, 7]. Since then [6], several retrospective studies have looked into clinical and genetic markers that are predictive of Ven‐HMA treatment response and post‐Ven‐HMA overall and relapse‐free survival (RFS) [4, 8, 9, 10]. The most recent publication, in this regard, highlighted a 4‐gene molecular signature for Ven‐HMA response and survival, based on the presence or absence of *TP53*, *KRAS*, *NRAS*, and *FLT3‐ITD* mutations [11]. Other studies have suggested different sets of genetic predictors of outcome, including *IDH2* ^MUT^, *NPM1* ^MUT^, *DDX41* ^MUT^, and adverse karyotype [4, 10, 12]. In the current study, we took advantage of a recently updated and expanded institutional database, in order to develop enhanced genetic risk models for treatment response and survival following Ven‐HMA therapy in ND‐AML. Two independent external patient cohorts were accessed for model validation.

## 2 Methods

Patients were retrospectively recruited from Mayo Clinic, USA (MN, AZ, FL), after Institutional Review Board approval and based on documentation of ND‐AML and treatment with at least one cycle of Ven‐HMA. All patients were treated outside clinical trials between November 2018 and May 2024 with follow‐up updated in July 2024. Cytogenetic and molecular studies (48‐gene panel) were performed through conventional karyotyping and next‐generation sequencing (NGS), respectively. *KMT2A* (also known as *MLL*) rearrangements (*KMT2Ar*) were confirmed by fluorescence in situ hybridization (FISH). Patients received either azacitidine 75 mg/m^2^ intravenously or subcutaneously days 1–7 or decitabine 20 mg/m^2^ intravenous days 1–5 plus Ven (median 200 mg; range 50–400 mg) by mouth daily for 7–28 days during the first cycle. Diagnosis, cytogenetic risk stratification, and response assessments were conducted according to the ELN 2022 criteria [13]. In the majority of cases, response was assessed after completion of one or two cycles, based on treating physician discretion. MRD was quantified using multiparameter flow cytometry with a minimum sensitivity of 0.01% and measured once CR or CRi with < 5% bone marrow blasts was documented. Relapse was defined by the emergence of ≥ 5% bone marrow or peripheral blood blasts, in patients with CR/CRi. RFS was calculated from the time of remission to relapse or last follow‐up/death and OS from the time of Ven‐HMA initiation to last‐follow up/death. During survival analysis, patients receiving allogeneic stem cell transplant (ASCT) were censored at the time of transplantation. Cox proportional hazard regression model was used to evaluate covariate associations with OS and RFS and the Kaplan–Meier method used to estimate RFS and OS. Risk models were developed using hazard ratio (HR)‐based risk point allocation and predictive accuracy was compared using Akaike Information Criterion (AIC) and area under the ROC curve (AUC). Statistical analyses were conducted using JMP Pro 18.0.0 software (SAS Institute, Cary, NC, USA).

## 3 Results

### 3.1 Patient Characteristics and Treatment Details

A total of 400 adult patients with ND‐AML were considered: median age 73 years (range: 19–98); 64% males; 95% white; 60% de novo; 22% secondary (post‐myelodysplastic syndrome [MDS], or myelodysplastic/myeloproliferative neoplasm [MDS/MPN]); and 18% therapy‐related. Treatment included a median of 4 cycles (range: 1–65) of Ven with decitabine (*n* = 265, 66%) or azacitidine (*n* = 148, 37%). Prior HMA exposure was documented in 30 (8%) patients. Cycle 1 Ven duration was 28 days (*n* = 237, 59%), 21 days (*n* = 59, 15%), 14 days (*n* = 63, 16%), or 7 days (*n* = 9, 2%). Key clinical and laboratory findings at time of treatment initiation are described in Table 1. ELN cytogenetic risk was evaluable in 398 cases and included favorable 2% (*n* = 7), intermediate 60% (*n* = 240), or adverse 38% (*n* = 151). 102 (26%) and 7 (2%) of cases harbored complex or monosomal karyotype and *KMT2Ar*, respectively. Recurrently mutated genes included *TP53* in 26% (101/394, 90% multi‐hit), *TET2* 19% (74/387), *RUNX1* 19% (76/392), *SRSF2* 18% (70/387), *ASXL1* 18% (70/387), *DNMT3A* 15% (57/392), *NPM1* 12% (49/394), *IDH2* 12% (48/393, 9% R140Q, 3% R172K), *FLT3‐ITD* 10% (40/397), *NRAS* 9% (34/392), *BCOR* 8% (32/387), *STAG2* 8% (22/376), *IDH1* 7% (26/393), *U2AF1* 6% (22/372), *SF3B1* 5% (19/376), *KRAS* 4% (15/392), and *DDX41* in 4% (14/376) of informative cases; 36% of *DDX41* ^MUT^ were confirmed as germline.

**TABLE 1:** Clinical characteristics at time of treatment with venetoclax and hypomethylating agent for 400 patients with newly diagnosed acute myeloid leukemia stratified by achievement of complete response with (CR) or without (CRi) count recovery.

Variables	All patients *N* = 400	Patients in CR/CRi *N* = 247 (62%)	Patients not in CR/CRi *N* = 153 (38%)	Univariate *p*‐value
Age in years, median (range)	73 (19–98)	73 (37–91)	73 (19–98)	0.85
Male, *n* (%)	256 (64)	152 (59)	104 (41)	0.19
Race, *n* (%)	**393**	**245**	**148**	0.68
White	375 (95)	235 (63)	140 (37)	
Black	9 (2)	6 (67)	3 (33)	
Asian	3 (1)	1 (33)	2 (67)	
Other	6 (2)	3 (50)	3 (50)	
AML type, *n* (%)				
De novo	239 (60)	157 (66)	82 (34)	0.07
Secondary	87 (22)	45 (52)	42 (48)	**0.03**
Therapy‐related	74 (18)	45 (62)	29 (38)	(Secondary vs. others)
Prior HMA, *n* (%)	30 (8)	5 (17)	25 (83)	**< 0.01**
Hemoglobin, g/dl, median (range)	8.5 (2.7–15.3)	8.7 (2.7–15.3)	8.2 (5.1–13)	0.05
Leukocyte count × 10^9^/L, median (range)	3.5 (0.4–243.8)	3.09 (0.4–200)	4.21 (0.5–243.8)	0.91
Platelet count × 10^9^/L, median (range)	53 (5–601)	62 (7–601)	40 (5–473)	**0.02**
Circulating blasts %, median (range)	11 (0–93)	11 (0–93)	12 (0–92)	0.37
Bone marrow blasts %, median (range)	43 (1–97)	43 (1–95)	43 (5–97)	0.89
ELN 2022 cytogenetic risk stratification, *n* (%)	**398**	**246**	**152**	
Favorable	7 (2)	5 (71)	2 (29)	
Intermediate	240 (60)	169 (70)	71 (30)	**< 0.01**
Adverse	151 (38)	72 (48)	79 (52)	
*KMT2A* rearrangement,^a^ *n* (%)	7/398 (2)	3/246 (43)	4/152 (57)	0.31
Mutations on NGS, evaluable, *n* (%)				
*TP53* (394)	101 (26)	46 (46)	55 (54)	**< 0.01**
*RUNX1* (392)	76 (19)	37 (49)	39 (51)	**< 0.01**
*TET2* (387)	74 (19)	50 (68)	24 (32)	0.23
*SRSF2* (387)	70 (18)	50 (71)	20 (29)	0.06
*ASXL1* (387)	70 (18)	43 (61)	27 (39)	0.99
*DNMT3A* (392)	57 (15)	44 (77)	13 (23)	**< 0.01**
*NPM1* (394)	49 (12)	41 (84)	8 (16)	**< 0.01**
*IDH2* (393)	48 (12)	37 (77)	11 (23)	**0.02**
*IDH1* (393)	26 (7)	20 (77)	6 (23)	0.09
*FLT3‐*ITD (397)	39 (10)	16 (41)	23 (59)	**< 0.01**
*NRAS* (392)	34 (9)	22 (65)	12 (35)	0.71
*BCOR* (387)	32 (8)	16 (50)	16 (50)	0.17
*U2AF1* (372)	22 (6)	12 (55)	10 (45)	0.47
*CEBPA* (*394*)	22 (6)	18 (82)	4 (18)	**0.04**
*CEBPA bZIP* (393)	12 (3)	10 (83)	2 (17)	0.10
*STAG2* (376)	22 (8)	17 (77)	5 (23)	0.11
*SF3B1* (376)	19 (5)	10 (53)	9 (47)	0.41
*KRAS* (392)	15 (4)	8 (53)	7 (47)	0.49
*DDX41* (376)	14 (4)	13 (93)	1 (7)	**< 0.01**
*PTPN11* (376)	12 (3)	7 (58)	5 (42)	0.81
*EZH2* (387)	11 (3)	4 (36)	7 (64)	0.09
*WT1* (387)	11 (3)	4 (36)	7 (64)	0.09
*SETBP1* (387)	11 (3)	5 (45)	6 (55)	0.27
*PHF6* (376)	10 (3)	6 (60)	4 (40)	0.91
*JAK2* (376)	9 (2)	7 (78)	2 (22)	0.30
*CBL* (375)	7 (2)	4 (57)	3 (43)	0.79
HMA used, *n* (%)				
Azacitidine	144 (36)	88 (62)	56 (38)	0.84
Decitabine	256 (64)	159 (62)	97 (38)	
Final dose of Venetoclax, mg, median (range)	200 (50–400)	200 (50–400)	200 (50–400)	0.29
Allogeneic transplant, *n* (%)	65 (16)	60 (92)	5 (8)	**< 0.01**

Abbreviations: ELN, European Leukemia Net; HMA, hypomethylating agent; NGS, next generation sequencing.

^a^ KMT2A‐PTD were not assessed.

### 3.2 Clinical and Genetic Predictors of Response

Overall, 153 (38%) patients achieved CR, and 94 (24%) CRi, resulting in CR or CRi in 247 (62%) of patients. CR/CRi rates were lower in patients with prior HMA exposure (17% vs. 65%; *p* < 0.01). MRD by multiparameter flow cytometry was undetected in 117 (70%) of 166 informative cases. Median time to CR/CRi was 1.3 months (range: 1–9) and median remission duration 6 months (range: 1–31). Table 1 compares the clinical and genetic profile of responders versus non‐responders. CR/CRi rates were inferior in patients with secondary AML (52% vs. 65%; *p* = 0.03). Considering genetic variables only, higher CR/CRi rates were seen with *NPM1* ^MUT^ (86% vs. 59%; *p* < 0.01), *IDH2* ^MUT^ (77% vs. 60%; *p* = 0.02; *IDH2* ^MUT^ 172 K vs. 140Q) (91% vs. 73%; *p* = 0.18), *DDX41* ^MUT^ (93% vs. 61%; *p* = 0.01), or *DNMT3A* ^MUT^ (77% vs. 59%; *p* < 0.01); *SRSF2* ^MUT^ (71% vs. 59%; *p* = 0.06) or *IDH1* ^MUT^ (77% vs. 61%; *p* = 0.09) displayed borderline significance. CR/CRi rates were lower in the presence of *TP53* ^MUT^ (45% vs. 67%; *p* < 0.01), *FLT3‐*ITD^MUT^ (41% vs. 64%; *p* = 0.01), or *RUNX1* ^MUT^ (49% vs. 65%; *p* < 0.01) or ELN 2022 adverse karyotype (48% vs. 71%; *p* < 0.01). Of note, CR/CRi rates were not influenced by the presence of *KRAS* ^MUT^ (53% vs. 62%; *p* = 0.49) or *NRAS* ^MUT^ (65% vs. 61%; *p* = 0.71) mutations, or *KMT2Ar* (43% vs. 62%; *p* = 0.31). In a separate analysis which excluded patients with prior HMA exposure, results were unchanged, except secondary AML did not impact response rates (CR/CRi; 65% vs. 66%; *p* = 0.87).

Table S1 lists predictors of treatment response based on univariate and multivariable analyses, inclusive of (i) mutations alone, (ii) mutations and karyotype, and (iii) mutations, karyotype, and clinical variables. In multivariable analysis that included both clinical and genetic variables, response rates were higher in the presence of *NPM1* ^MUT^ (*p* < 0.01; OR: 0.41) and lower in the presence of secondary AML (*p* = 0.03; OR: 1.8), adverse karyotype (*p* < 0.01; OR: 2.3), *TP53* ^MUT^ (*p* = 0.04; OR: 1.9), *FLT3‐ITD* ^MUT^ (*p* < 0.01; OR: 4.8), or *RUNX1* ^MUT^ (*p* < 0.01; OR: 2.4). In multivariate analysis of karyotype and mutations, independent predictors of superior response included *NPM1* ^MUT^ (*p* < 0.03; OR: 0.53) and inferior response adverse karyotype (*p* < 0.01; OR: 2.3), *TP53* ^MUT^ (*p* = 0.04; OR: 1.9), *FLT3‐ITD* ^MUT^ (*p* < 0.01; OR: 4.8), or *RUNX1* ^MUT^ (*p* < 0.01; OR: 2.4). The presence of *TP53* ^MUT^ did not appear to influence response rates among patients with either ELN defined non‐adverse (CR/CRi; 57% vs. 71% in *TP53* ^MUT^ vs. *TP53* ^WT^; *p* = 0.45) or adverse karyotype (CR/CRi, 45% vs. 53% in *TP53* ^MUT^ vs. *TP53* ^WT^; *p* = 0.34). Similar findings were obtained when complex or monosomal karyotype was considered.

**FIGURE 1:**
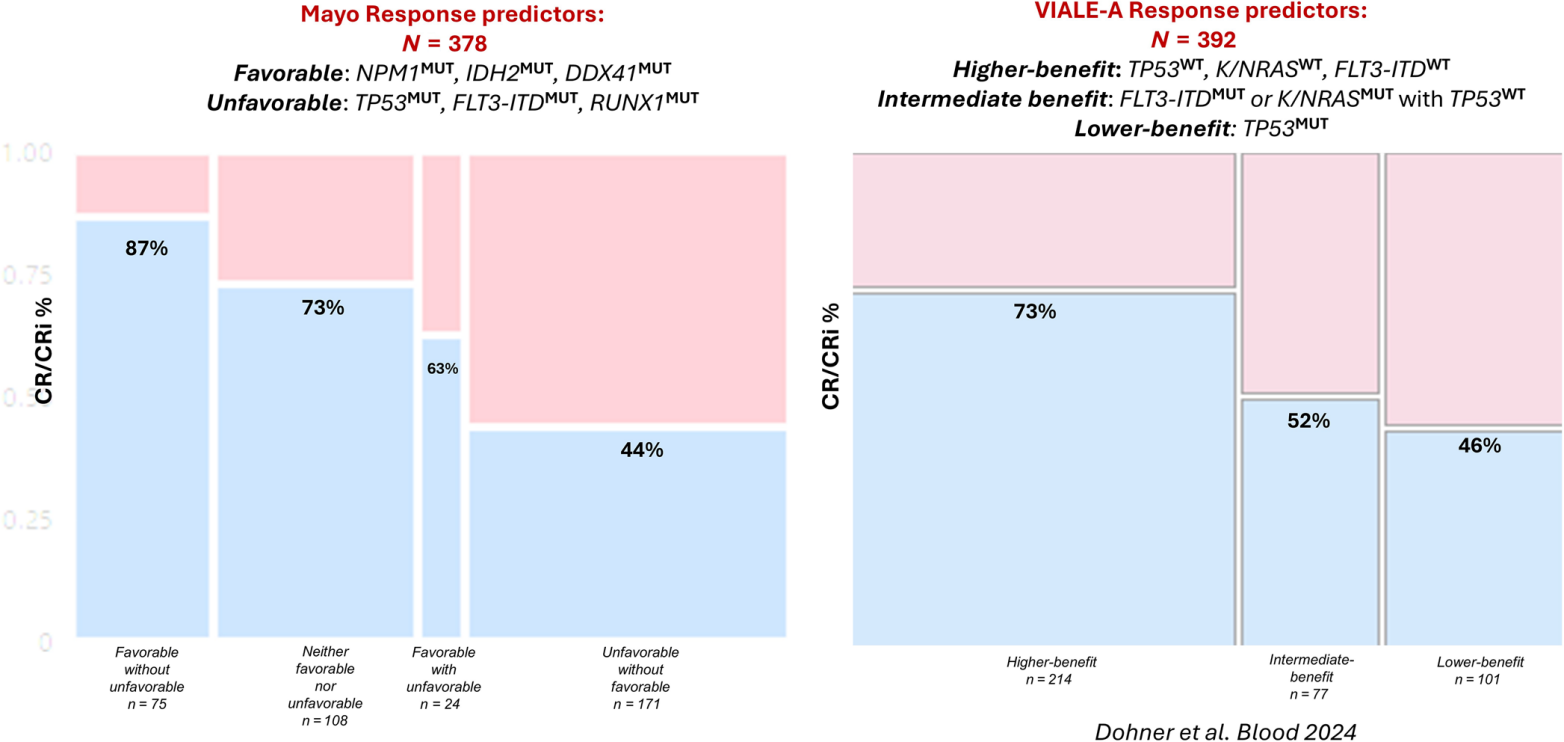
Genetic signature for response in newly diagnosed acute myeloid leukemia treated with venetoclax and hypomethylating agent. [Color figure can be viewed at wileyonlinelibrary.com]

In multivariable analysis limited to mutations, *NPM1* ^MUT^, *IDH2* ^MUT^, and *DDX41* ^MUT^ were identified as positive and *TP53* ^MUT^, *FLT3‐ITD* ^MUT^, and *RUNX1* ^MUT^ as negative predictors of treatment response. CR/CRi rate was highest at 87% in patients harboring one or more *favorable* mutations (*NPM1*, *IDH2*, *DDX41*) and no *unfavorable* mutation (*TP53*, *FLT3‐*ITD, *RUNX1*). CR/CRi rate was lowest at 44% in patients with at least one *unfavorable* mutation and no *favorable* mutation (*p* < 0.01). CR/CRi rates were similar in patients with neither *favorable* nor *unfavorable* mutations versus those harboring *favorable* with *unfavorable* mutations (73% vs. 63%; *p* > 0.1) (Figure 1). Application of the VIALE‐A 4‐gene signature for Ven‐HMA response yielded CR/CRi rates of 46% in the presence of *TP53* ^MUT^ (lower‐benefit group), 52% in *TP53* ^WT^ and presence of either *FLT3‐ITD* ^MUT^ or *KRAS/NRAS* ^MUT^ (intermediate group) and 73% in the absence of *TP53* ^MUT^, *KRAS/NRAS* ^MUT^, or *FLT3‐ITD* ^MUT^ (higher‐benefit group) (Figure 1); in particular, CR/CRi rates were found to be similar for intermediate vs. lower‐benefit groups (52% vs. 46%; *p* = 0.39). Among *NPM1* ^MUT^ and *IDH2* ^MUT^ cases, presence of *FLT3‐ITD* ^MUT^ and *K/NRAS* ^MUT^ did not appear to significantly influence response rates; among 48 patients with *IDH2* ^MUT^, *FLT3‐ITD* ^MUT^ was present in 5 (10%) while 2 (4%) harbored *K/NRAS* ^MUT^; CR/CRi rate was 60% versus 79% (presence vs. absence of *FLT3‐ITD* ^MUT^ *p* > 0.1) and 100% versus 76% (presence vs. absence of *K/NRAS* ^MUT^) (*p* > 0.1). Among 49 *NPM1* ^MUT^ patients, 11 (22%) harbored *FLT3‐ITD* ^MUT^ (CR/CRi; 73% vs. 87%; *p* = 0.29), and 13 (27%) *K/NRAS* ^MUT^ (CR/CRi; 85% vs. 83%; *p* = 0.88). Of note, *DDX41* mutated patients did not harbor either *FLT3‐ITD* ^MUT^ or *K/NRAS* ^MUT^.

CR rates in comparison to CRi were lower in *FLT3‐ITD* (38% vs. 63%; *p* = 0.04), *SRSF2* (46% vs. 54%; *p* = 0.016), and *ASXL*1 mutated cases (42% vs. 58%; *p* < 0.01). A trend for lower CR versus CRi rates was observed in *IDH2* (49% vs. 51%; *p* = 0.09), *KRAS* (33% vs. 68%; *p* = 0.16), and *RUNX1* mutated (49% vs. 51%; *p* = 0.09); on the other hand, CR rates were significantly higher in *DDX41* mutated (92% vs. 8%; *p* < 0.01), and non‐significantly higher CR versus CRi rates were observed in *TP53* mutated cases (69% vs. 30%; *p* = 0.19).

### 3.3 Clinical and Genetic Predictors of Relapse

Relapse was documented in total of 99 (40%) patients who achieved CR (39%) or CRi (41%) after a median remission duration of 6 months (1–31). Patients that relapsed within the first‐year versus those that did not were more likely to be males (75% vs. 55%), MRD positive (44% vs. 24%), and harbor *TP53* ^MUT^ (23% vs. 16%). Univariate analysis for RFS identified male gender (12 vs. 29 months; *p* = 0.02), secondary AML (9 vs. 18 months; *p* < 0.01), adverse karyotype (9 vs. 22 months; *p* < 0.01), *TP53* ^MUT^ (6 vs. 18 months, *p* < 0.01), and MRD positive remission (13 months vs. not reached; *p* < 0.01) as risk factors for relapse; CRi versus CR were borderline significant (15 vs. 17 months; *p* = 0.09); on the other hand, *IDH2* ^MUT^ (not reached vs. 15 months; *p* < 0.01) were associated with a lower risk of relapse.

Multivariable analysis inclusive of genetic variables and MRD status confirmed *TP53* ^MUT^ (HR: 2.4) and MRD positive remission (HR: 2.0) as *unfavorable* predictors of relapse (Table 2). Subsequently, in a three‐tiered relapse prediction model, RFS was significantly inferior in the presence of two risk factors (*TP53* ^MUT^, MRD positive remission) (*n* = 11, median 4 months), vs. one (*n* = 57, median 16 months) versus none of the risk factors (*n* = 96, median not reached) (*p* < 0.01); 1‐year cumulative incidence of relapse was 84%, 45%, and 28% in the presence of two, one and none of the risk factors, respectively (Figure S1).

**TABLE 2:** Predictors of overall survival and relapse‐free survival in 400 patients with newly diagnosed acute myeloid leukemia receiving venetoclax plus hypomethylating agent therapy.

Variables	Overall survival transplant‐censored				Relapse‐free survival Relapse, *N* = 166	
	Univariate *p*‐value HR (95% CI)	Multivariate with pre‐treatment variables clinical + genetics *p*‐value HR (95% CI)	Multivariate with genetic variables only *p*‐value HR (95% CI)	Multivariate with Response *p*‐value HR (95% CI)	Univariate *p*‐value HR (95% CI)	Multivariate with genetic variables *p*‐value HR (95% CI)
Age	0.83				0.17	
Gender	**< 0.01** 1.6 (1.2–2.2) Male vs. female	**< 0.01** 2.1 (1.5–2.9)		**< 0.01** 2.2 (1.6–2.9) Male vs. female	**0.02** 1.7 (1.1–2.6) Male vs. female	
Secondary AML	**< 0.01** 1.5 (1.2–1.9) Presence vs. Absence	**< 0.01** 1.6 (1.2–2.2)		0.05	**< 0.01** 1.8 (1.2–2.9) Presence vs. Absence	
ELN 2022 adverse karyotype	**< 0.01** 2.6 (2.1–3.4) Presence vs. Absence	**< 0.01** 1.9 (1.3–2.8)	**< 0.01** 1.8 (1.2–2.6)	**< 0.01** 1.8 (1.3–2.7)	**< 0.01** 2.3 (1.5–3.5) Presence vs. Absence	0.31
*KMT2A* rearrangement	**< 0.01** 5.5 (2.4–12.5)	**< 0.01** 7.9 (3.3–19.1)	**< 0.01** 4.7 (2.0–11.1)	**< 0.01** 7.8 (3.3–18.6)	0.15	
*TP53* mutation	**< 0.01** 2.6 (1.9–3.5) Presence vs. Absence	**< 0.01** 1.9 (1.3–2.8)	**< 0.01** 1.7 (1.2–2.6)	0.07	**< 0.01** 2.4 (1.5–2.9) Presence vs. Absence	**< 0.01** 2.4 (1.3–4.4)
*IDH2* mutation	**< 0.01** 2.6 (1.5–4.4) Absence vs. Presence	**0.01** 2.0 (1.1–3.4)	**< 0.01** 2.1 (1.2–3.6)	**< 0.01** 2.4 (1.4–4.1)	**< 0.01** 2.6 (1.2–5.7) Absence vs. Presence	0.10
*IDH1* mutation	**0.01** 2.1 (1.1–4.1) Absence vs. Presence	0.19	0.08	0.66	0.68	
*NPM1* mutation	**0.06** 1.5 (0.98–2.3) Absence vs. Presence	0.86			0.13	
*DNMT3A* mutation	**0.01** 1.6 (1.1–2.4) Absence vs. Presence	0.48	0.21	0.45	0.51	
*FLT3‐*ITD mutation	0.63				0.31	
*RUNX1* mutation	0.73				0.62	
*DDX41* mutation	**0.02** 3.2 (1.2–8.7) Absence vs. Presence	0.07	0.08	0.09	0.83	
*KRAS* mutation	**0.05** 1.7 (1.0–3.2) Presence vs. Absence	**0.02** 2.1 (1.2–3.9)	**< 0.01** 2.2 (1.2–4.0)	0.15	0.81	
*NRAS* mutation	0.46				0.25	
CR/CRi	**< 0.01** 5.4 (3.5–6.8) Absence vs. Presence			**< 0.01** 5.2 (3.9–6.9)		
MRD positive vs. negative	0.31				**< 0.01** 2.1 (1.3–3.6) Presence vs. Absence	**< 0.01** 2.0 (1.2–3.4)

Abbreviations: CR, complete remission; CRi, CR with incomplete count recovery; ELN, European Leukemia Net; MRD, measurable residual disease.

50 of 99 (65%) patients received salvage therapy which included cladribine‐cytarabine‐Ven (*n* = 19), intensive induction chemotherapy (*n* = 9), FLT3 inhibitors (*n* = 5), Ven‐HMA‐FLT3 inhibitor (*n* = 1), IDH1/2 inhibitors (*n* = 5), glasdegib‐cytarabine (*n* = 4), gemtuzumab (*n* = 3), lenalidomide (*n* = 1), or investigational therapies (*n* = 3).

### 3.4 Risk Factor Analysis for Overall Survival and Development of a New Genetic Risk Model

After a median follow‐up of 10.5 months (range 0.5–66), 248 death (62%; including 62 within 3 months of treatment initiation) and 65 ASCT (16%; including 60 in CR/CRi) were documented. Median OS for the cohort was 12.6 months; 30‐day and 60‐day mortality rates were 4% and 11%, respectively. Table 2 outlines clinical and genetic variables found to affect transplant‐censored OS in univariate and multivariate analyses. Univariate analysis for transplant‐censored OS disclosed superior survival in presence of CR/CRi (median 21 vs. 3.6 months; HR: 5.4), *IDH2* ^MUT^ (not reached vs. 11 months; HR: 2.6), *IDH1* ^MUT^ (not reached vs. 11.9 months; HR: 2.1), *DNMT3A* ^MUT^ (18.2 vs. 10.9 months; HR: 1.6), or *DDX41* ^MUT^ (25.8 vs. 11.9 months; HR: 3.2). By contrast, male gender (9.7 vs. 19.2 months; HR: 1.6), secondary AML (9.7 vs. 14.1 months; HR: 1.5), adverse karyotype (7.4 vs. 18.6 months; HR: 2.6), *KMT2Ar* (2.5 vs. 12.9 months; HR: 5.5), *TP53* ^MUT^ (6.2 vs. 16.6 months; HR: 2.6), or *KRAS* ^MUT^ (5.7 vs. 12.7 months; HR: 1.7) were associated with inferior survival (Table 2). Among *IDH2* mutated patients, survival was superior in patients with *IDH2* ^MUT^ R172K versus R140Q mutations (not reached vs. 19.2 months; *p* = 0.02). OS was similar in patients achieving CR versus CRi (23 vs. 19 months; *p* = 0.44). In a separate analysis which excluded patients with prior HMA exposure, results were unchanged, except secondary AML did not impact OS (*p* = 0.21).

**FIGURE 2:**
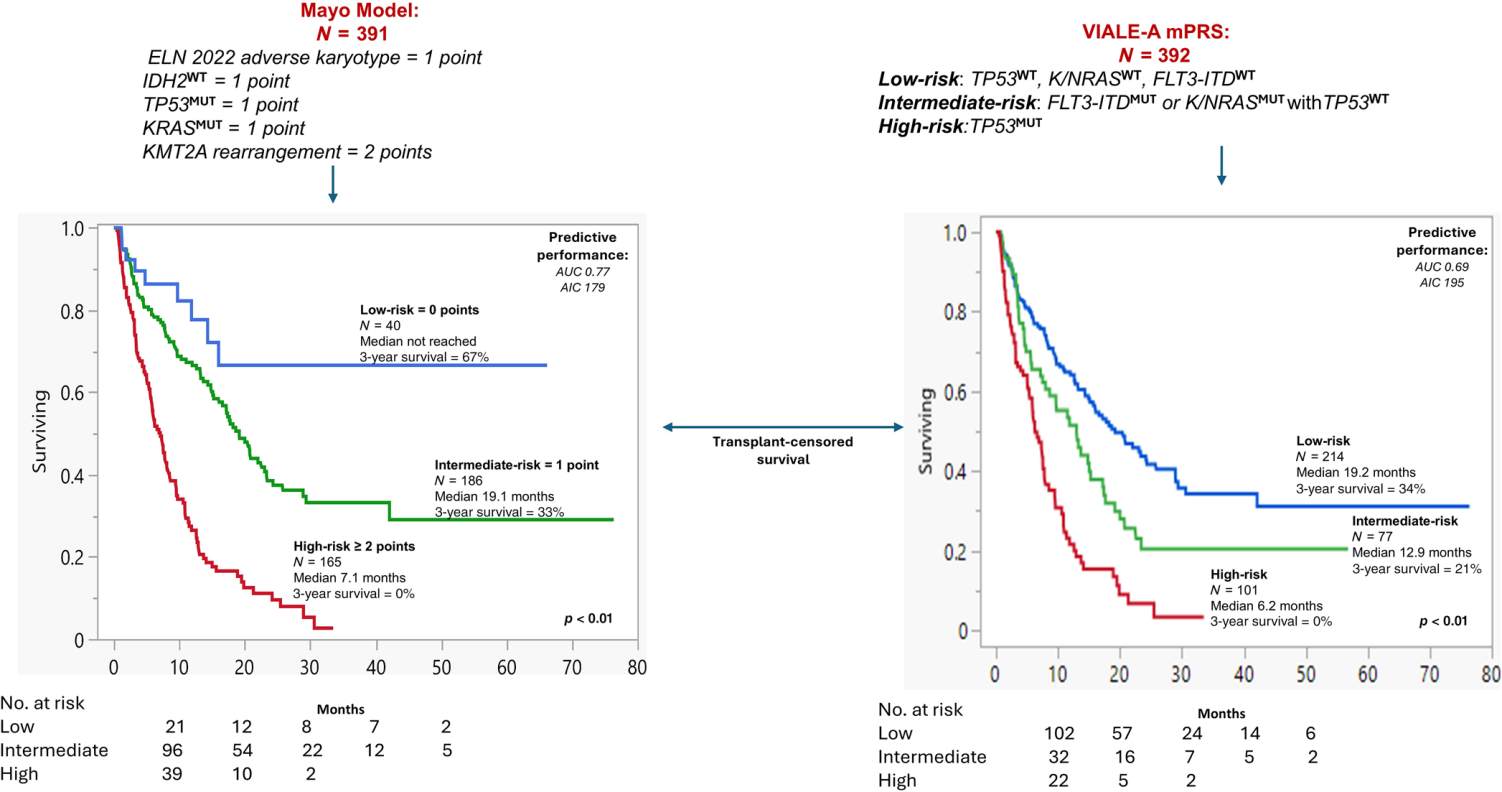
Mayo genetic risk model versus European leukemia net (ELN) 2024 model in newly diagnosed acute myeloid leukemia treated with venetoclax and hypomethylating agent. [Color figure can be viewed at wileyonlinelibrary.com]

Multivariable analysis of clinical and genetic variables confirmed independent prognostic significance for male gender, secondary AML, adverse karyotype, *KMT2Ar*, *TP53* ^MUT^, *KRAS* ^MUT^ and *IDH2* ^WT^, with respective HRs (95% CI) of 2.1 (1.5–2.9), 1.6 (1.2–2.2), 1.9 (1.3–2.8), 7.9 (3.3–19.1), 1.9 (1.3–2.8), 2.2 (1.2–4.0), and 2.0 (1.1–3.4). Multivariable analysis limited to genetic variables only identified four independent risk factors for OS: HR (95% CI) were 1.8 (1.2–2.6) for adverse karyotype, 4.7 (2.0–11.1) for *KMT2Ar*, 1.7 (1.2–2.6) for *TP53* ^MUT^, 2.6 (1.4–4.7) for *KRAS* ^MUT^, and 2.1 (1.2–3.6) for *IDH2* ^WT^. HR‐weighted scoring led to respective assignment of one adverse point each for ELN adverse karyotype, *TP53* ^MUT^, *KRAS* ^MUT^, *IDH2* ^WT^, and two adverse points for *KMT2Ar*, which resulted in a three‐tiered risk stratification: low‐risk (0 points, *n* = 40), intermediate (1 point, *n* = 186), and high‐risk (≥ 2 points, *n* = 165). Figure 2 displays survival data using the Mayo genetic risk model with respective median survival (3‐year survival rate) of median not reached (67%), 19.1 months (30%), and 7.1 months (0%) (*p*‐values < 0.01 for all comparisons). Among seven patients with *KMT2Ar*, one patient was upgraded from intermediate to high‐risk and six from high to very‐high risk with median OS of 2.2 months for very‐high risk patients (≥ 4 points, *n* = 6) (Figure S2).

### 3.5 Overall Survival Analysis in Treatment Responders and Development of a Response‐Stratified Survival Model

**FIGURE 3:**
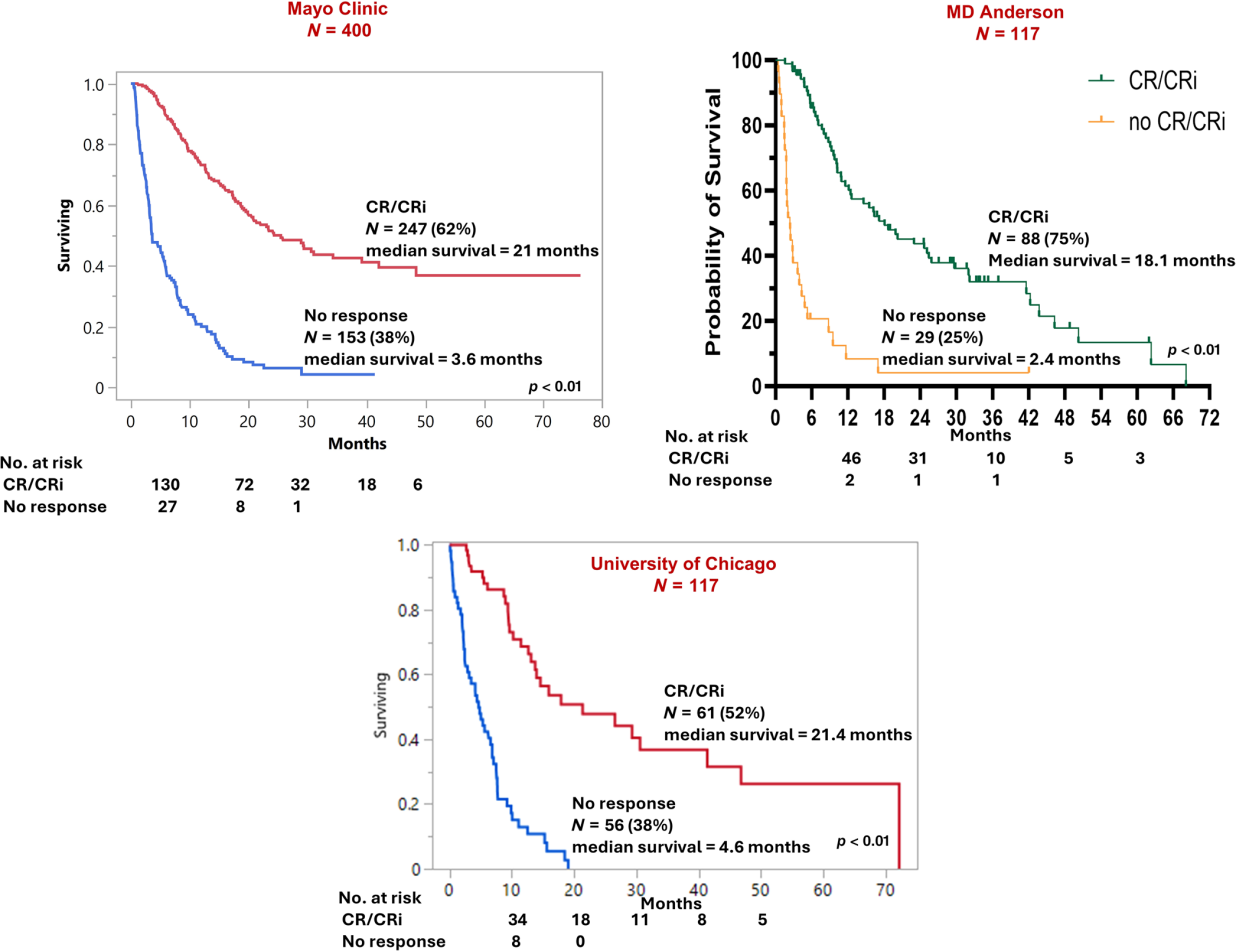
Overall survival in patients with newly diagnosed acute myeloid leukemia treated with venetoclax and hypomethylating agent, stratified by response. [Color figure can be viewed at wileyonlinelibrary.com]

**FIGURE 4:**
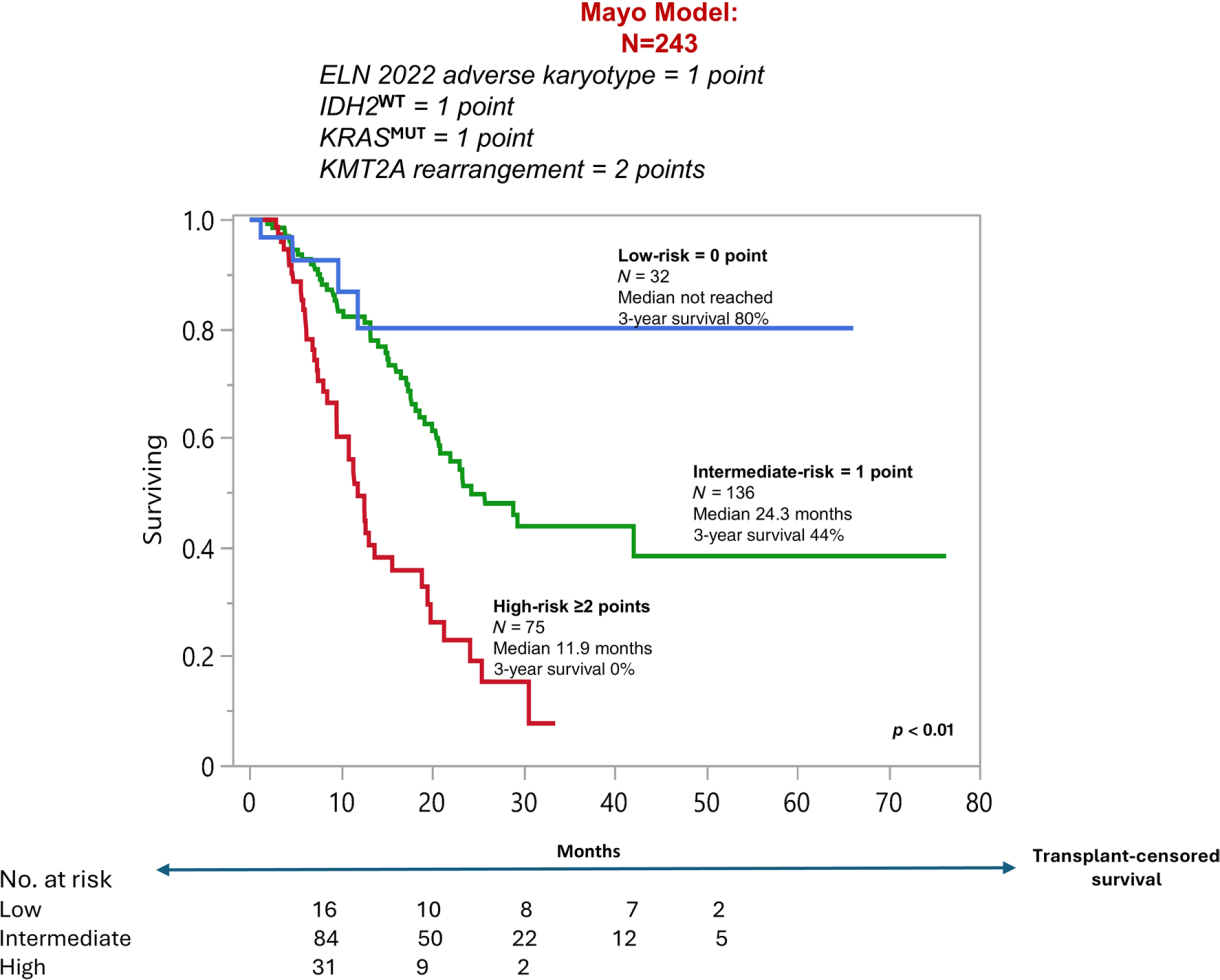
Mayo genetic risk model in newly diagnosed acute myeloid leukemia treated with venetoclax and hypomethylating agent and achieving complete remission with (CR) or without (CRi) count recovery. [Color figure can be viewed at wileyonlinelibrary.com]

Achievement of CR/CRi was the strongest predictor of survival with median (3‐year survival rate) of 3.6 versus 21 months (4% vs. 43%) and HR 5.4 in the absence versus presence of CR/CRi (Figure 3). Accordingly, a separate analysis of risk factors for survival was performed in treatment responders (Table S2). As expected, survival was superior in MRD negative versus positive cases (39 vs. 23 months; *p* = 0.09). In addition, in univariate analysis, *IDH2* ^MUT^ was associated with superior OS (median not reached vs. 20.5 months). Male gender (median 17.6 vs. 29.2 months), *TP53* ^MUT^ (median 11.9 vs. 24.3 months), *KRAS* ^MUT^ (median 11.4 vs. 22 months), adverse karyotype (median 12.6 vs. 29.4 months), and *KMT2Ar* (median 5.2 vs. 22 months) were associated with inferior OS. Multivariable analysis of genetic variables resulted in HRs (95% CI) of 2.3 (1.5–3.6) for adverse karyotype, 7.5 (1.7–32.7) for *KMT2Ar*, 3.4 (1.4–8.5) for *KRAS* ^MUT^, and 2.8 (1.2–6.5) for *IDH2* ^WT^; *TP53* ^MUT^ was no longer significant (*p* = 0.14). Accordingly, a response‐stratified Mayo genetic risk model was generated with assignment of one point each to adverse karyotype, *KRAS* ^MUT^ and *IDH2* ^WT^ and two adverse points for *KMT2Ar*: high risk ≥ 2 points (*n* = 75; median 11.9 months; 3‐year survival, 0%); intermediate risk = 1 point (*n* = 136; median 24.3 months; 3‐year survival, 44%); and low‐risk = 0 points (*n* = 32; median not reached; 3‐year survival, 80%; *p* < 0.01) (Figure 4). 3‐year survival for high‐risk with or without ASCT was 42% versus 0% (*p* < 0.01); intermediate 72% versus 43% (*p* = 0.06); and low‐risk 88% versus 78% (*p* = 0.53).

### 3.6 Comparison of the Mayo Prognostic Model and ELN 2024 Genetic Risk Model

Figure 2 illustrates performance comparisons between the Mayo and ELN 2024 genetic risk models. Based on the ELN 2024 genetic risk model, low‐risk (*TP53* ^WT^, *K/NRAS* ^WT^, *FLT3‐ITD* ^WT^), intermediate (*FLT3‐ITD* ^MUT^ or *K/NRAS* ^MUT^ with *TP53* ^WT^) and high‐risk (*TP53* ^MUT^) groups had respective median survival (3‐year survival rate) of 19.2 (34%), 12.9 months (21%), and 6.2 months (0%); intermediate versus low risk (*p* = 0.01, HR: 1.5) and high versus intermediate (*p* < 0.01, HR: 1.9). On the other hand, the Mayo genetic risk model provided a more pronounced distinction between high versus intermediate (7.1 vs. 19.1 months; *p* < 0.01, HR 2.7) and intermediate versus low‐risk categories (19.1 months vs. not reached; *p* = 0.02; HR 2.2). In addition, comparison of AUC/AICc estimates for the Mayo and ELN 2024 models considering 3‐year survival probability demonstrated superior performance of the Mayo model (AUC 0.77, AICc 179) versus ELN 2024 model (AUC 0.69, AICc 195).

### 3.7 Validation of the Mayo Genetic Risk Model

The Mayo genetic risk model was validated by using an MD Anderson Cancer Center cohort (MDACC) of 117 ND‐AML patients (median age 73; years, 61% males) receiving Ven‐HMA; 53 (45%) of patients harbored ELN 2022 adverse karyotype; 3 (3%) with *KMT2Ar*; mutations involved *TP53* (35%), *NPM1* (22%), *IDH2* (15%), *RUNX1* (12%), *IDH1* (10%), *FLT3‐ITD* (3%), *DDX41* (3%). In this cohort, CR/CRi was achieved in 88 (75%) of patients (Table S4). Application of the Mayo genetic risk model to the MDACC cohort yielded median OS of 43.7 (0 points, *n* = 13), 19.9 months (1 point, *n* = 45), and 6.6 months (≥ 2 points, *n* = 59) (*p* < 0.01). In addition, implementation of the response‐stratified Mayo risk model which incorporated adverse karyotype, *KMT2Ar*, *KRAS* ^MUT^, and *IDH2* ^WT^ also disclosed similar results (Figure S3).

In a second external cohort of 117 ND‐AML patients (median age 74 years, 60% males) treated with Ven‐HMA at the University of Chicago (UOC), 45 of 113 (40%) of informative cases harbored adverse karyotype; 2 (2%) with *KMT2Ar*; mutations involved *TP53* (30%), *RUNX1* (22%), *IDH1/2* (11%), *NPM1* (10%), *FLT3‐ITD* (9%), *DDX41* (3%). CR/CRi was documented in 61 (52%) of patients. In multivariable analysis which included relevant clinical and genetic variables, CR/CRi was the only independent predictor of OS (*p* < 0.01, HR: 5.6) (Table S5).

## 4 Discussion

Ven‐HMA is currently the standard‐of‐care for patients with ND‐AML who are elderly and/or unfit for intensive induction chemotherapy [3, 14]. AML is a genetically heterogeneous disease with highly variable patient outcomes following Ven‐HMA therapy [4, 5, 10]. Accordingly, prognostic information including estimation of the likelihood of response, disease relapse, and chances of survival are instrumental in the decision‐making process. While previous studies have investigated the prognostic relevance of genetic factors in the context of Ven‐HMA therapy [8, 11], none had accounted for response to treatment. The current study defines four distinct molecular signatures of treatment response to Ven‐HMA: (i) *NPM1* ^MUT^, *IDH2* ^MUT^, or *DDX41* ^MUT^ with *TP53* ^WT^, *RUNX1* ^WT^, and *FLT3‐ITD* ^WT^ (CR/CRi, 87%), (ii) *NPM1* ^WT^, *IDH2* ^WT^, *DDX41* ^WT^, *TP53* ^WT^, *RUNX1* ^WT^, *FLT3‐ITD* ^WT^ (CR/CRi, 73%), (iii) *NPM1* ^MUT^, *IDH2* ^MUT^, or *DDX41* ^MUT^ with *TP53* ^MUT^, *RUNX1* ^MUT^, or *FLT3‐ITD* ^MUT^(CR/CRi, 63%), and (iv) *TP53* ^MUT^, *RUNX1* ^MUT^, or *FLT3‐ITD* ^MUT^ with *NPM1* ^WT^, *IDH2* ^WT^, or *DDX41* ^WT^ (CR/CRi, 44%). By contrast, in a pooled analysis of 279 patients treated with Ven‐azacitidine in the phase 3 VIALE‐A (NCT02993523) and phase 1b study (NCT02203773), CR/CRi rates were 77.2% in *TP53* ^WT^, *K/NRAS* ^WT^, and *FLT3‐ITD* ^WT^ (higher‐benefit group), 59.2% in the presence of *FLT3‐ITD* ^MUT^ or *K/NRAS* ^MUT^ with *TP53* ^WT^ (intermediate‐benefit group) and 47.6% in the presence of *TP53* ^MUT^ (lower‐benefit subgroup) [11]. When applied to the Mayo cohort, the VIALE‐A molecular subgroups resulted in respective CR/CRi rates of 73%, 52% and 46%, for higher, intermediate, and lower‐benefit groups, with marginal distinction between intermediate and lower‐benefit groups in terms of response. Interestingly, in the VIALE‐A molecular prognostic risk score (mPRS), *NPM1* ^MUT^ and *IDH2* ^MUT^, which have previously shown superior response to Ven‐HMA [4, 10, 15], were not found to impact response in the context of *TP53* ^MUT^, *K/NRAS* ^MUT^, *FLT3‐ITD* ^MUT^, *DDX41* ^MUT^ which has also been associated with favorable response to Ven‐HMA [12, 16], was not analyzed in the VIALE‐A mPRS.

*TP53* mutations are known to cluster with adverse karyotype, which was the case in 93% of *TP53* ^MUT^ patients in the current study; however, *TP53* ^MUT^ did not appear to influence response rates, independent of karyotype. On the other hand, presence of adverse or complex/monosomal karyotype was independently associated with inferior response (CR/CRi 48% vs. 71% with or without adverse karyotype, and 50% versus 67% with or without complex/monosomal karyotype; *p* < 0.01). In a separate analysis of the VIALE‐A study, including patients with poor‐risk cytogenetics plus *TP53* ^MUT^, Ven‐azacitidine improved remission rates but not duration of remission or OS compared with azacitidine alone; moreover, remission and OS rates were higher in patients with poor‐risk cytogenetics and *TP53* ^WT^ [17].

The vulnerability of *IDH2* mutations to Ven‐HMA therapy makes a case for its upfront use; in a multicenter study of 151 patients ≥ 60 years with *IDH1/2* mutated AML (90 *IDH2* mutated), receiving Ven‐HMA or intensive chemotherapy, CR/CRi (67% vs. 67%) and overall survival rates (2 year survival; 49% vs. 38%) were similar after adjusting for baseline patient characteristics [18]. Unlike the VIALE‐A mPRS, the unfavorable prognosis typically associated with *RAS* mutations was limited to *KRAS* as opposed to *NRAS* mutations.

Genetic risk factors which influenced survival in the current study included ELN‐defined adverse karyotype, *KMT2Ar*, *TP53* ^MUT^, *KRAS* ^MUT^, and *IDH2* ^WT^, which were accordingly incorporated into a pre‐treatment genetic risk model. Importantly, our study illustrates the prognostic value of CR/CRi which consistently overshadowed pre‐treatment risk variables in predicting survival in all three study cohorts in the current study (Mayo, MDACC, and UOC) and therefore allowed for a novel response‐stratified risk model inclusive of ELN‐defined adverse karyotype, *KMT2Ar*, *KRAS* ^MUT^, and *IDH2* ^WT^. It is to be noted that the inclusion of response status in survival analysis resulted in the loss of prognostic impact from *TP53* ^MUT^. Both pre‐treatment and response‐stratified genetic models were validated by an external cohort and are amenable to further refinements with additional MRD information.

It is important to recognize the key differences between the Mayo prognostic model and the VIALE‐A mPRS. Unlike the case in our study, presence of *IDH2* ^MUT^ was not found to be independently prognostic in the ELN model and was reportedly identified in all three risk categories; median OS for *IDH2* ^MUT^ patients was 36.9 months versus 12.2 months in the higher and intermediate‐benefit groups, respectively [11]. Furthermore, in multivariable analysis of clinical and genetic variables in the VIALE‐A cohort, only genetic variables (*TP53* ^MUT^, *KRAS* ^MUT^, *NRAS* ^MUT^, and *FLT3‐ITD* ^MUT^) were significant, whereas age ≥ 75 years, male gender, secondary AML, baseline blast counts, and ECOG performance status were not [11]. In the current study, the prognostic impact of adverse karyotype was independent of mutations; additionally, clinical variables of independent prognostic relevance included male gender and secondary AML. The association of male gender with inferior survival has been recently reported in older intensively treated patients with AML [19]. The above discrepancies between our findings and the VIALE‐A mPRS might have stemmed from differences in patient population with inclusion of cases with prior HMA exposure; additionally, all survival analyses in the current study accounted for ASCT.

Similar to the observation in the current study, a recent report by the BEAT‐AML investigators which included 595 patients with ND‐AML treated with a number of lower‐intensity therapies, including Ven‐HMA, identified *IDH2* ^MUT^ as an independent favorable prognostic factor, and *KRAS* ^MUT^, *MLL2* (*KMT2D*)^MUT^, and *TP53* ^MUT^ as unfavorable [20]. Based on these findings, a BEAT‐AML 2024 risk model was proposed with favorable, intermediate, and adverse‐risk groups with 2‐year OS: 48% versus 33% versus 11%, respectively, *p* < 0.01. Subsequently, this model was applied to patients receiving Ven‐HMA; risk group allocation included favorable (*n* = 49), intermediate (*n* = 83), and adverse‐risk (*n* = 70), with a significant difference between favorable and intermediate‐risk (*p* = 0.03); however, difference between intermediate and adverse risk groups was found to be marginal (OS and *p*‐value not provided) [20]. It is worthy to note that clinical variables, karyotype and ASCT, did not appear to be factored into the survival analysis [20].

The current study confirms that AML with *KMT2Ar* has adverse outcomes. In an MDACC study including 172 adult patients with *KMT2Ar* AML compared to 522 age‐matched AML patients with normal karyotype treated with high or low‐intensity chemotherapy regimens, the former had significantly inferior OS (median OS of 0.9 years vs. 2.1 years and 5‐year OS of 20% vs. 34%; *p* < 0.01), which was improved with ASCT (median 10.4 years and 5‐year OS 52%) [21].

Limitations of the current study include heterogeneous duration of venetoclax use and variability in timing of response assessment. The above‐discussed information underlines the challenges in prognostic factor assessments for Ven‐HMA‐treated ND‐AML. Methodological descriptions in some published studies were not always clear and established prognostic factors were not always considered in the statistical analysis, precluding comparison of study results. Nonetheless, the current study demonstrated superior model performance by the Mayo genetic risk model, compared to the recently published ELN 2024 risk model and also provides a novel response‐based risk stratification. The overarching value of such a model is consistent with established knowledge regarding the value of CR/CRi in predicting survival outcomes in ND‐AML treated with both intensive and less‐intensive therapies [10, 22, 23]. Going forward, incorporation of MRD information is likely to further enhance prognostic assessments [24, 25].

## Author Contributions

N.G., A.T. designed the study, collected data, performed analysis and wrote the paper. A.E., F.A., I.M.J., MA collected data. K.M., A.A.‐K., H.B.A., K.H.B., M.E., A.M., A.M., A.N.S., M.H., M.R.L., W.H., M.S., M.M.P., A.P., T.B., H.M., J.A., J.P., L.S., N.K., C.A. contributed patients. S.Y., A.S., E.D., A.A.P. collected data and contributed patients. A.B. collected data, performed analysis and contributed patients. J.S., A.B., C.D., T.K. contributed patients.

## Ethics Statement

IRB approval obtained.

## Consent

Waived due to minimal risk research.

## Conflicts of Interest

M.R.L: Research support from Abbvie, Astellas, Amgen, Actinium, Pluristem, Sanofi. Speaker's Bureau for Amgen, Beigene. Data Safety Monitoring Committee for Biosight. N.G.: Advisory Board for DISC medicine and Agios. M.S.: Research funding to the institution from Astellas, Celgene, and Marker Therapeutics. M.M.P.: Member of the board of directors or advisory committees of Stemline Therapeutics and received research funding from Kura Oncology. A.A.P.: Research funding from Pfizer, Kronos Bio, Sumitomo; honoraria from AbbVie, Sobi, Bristol Myers Squibb. ED: Honoraria from AbbVie. C.D.: Consultant/Advisory Boards: Abbvie, AstraZeneca, Astellas, BMS, Genentech, GenMab, GSK, Notable Labs, Rigel, Ryvu, Schrodinger, Servier. C.D.: is supported by the LLS Scholar in Clinical Research Award. T.K.: Grant support: BMS, Abbvie, Amgen, Ascentage Pharma Group, Astellas Pharma, DrenBio, Astex, AstraZeneca, BMS, Celgene, Incyte, Cellenkos, Cyclacel, Delta‐Fly pharma, Genentech, Genfleet, Glycomimetics, Iterion, Janssen, Jazz Pharmaceuticals, Pfizer, Pulmotect, Regeneron, SELLAS. Consulting fees: AbbVie, Agios, Daiichi Sankyo, Genentech, Genzyme, Jazz Pharmaceuticals, Liberum, Novartis, Pfizer, PinotBio, Pukmotect, Sanofi‐Aventis, Servier. Payment or honoraria: AbbVie, Agios, Daiichi Sankyo, DAVA Oncology, Delta‐Fly, DrenBio, Genentech, Genfleet, Genzyme, Jazz Pharmaceuticals, Liberum, Novartis, Pfizer, Rigel, Sanofi‐Aventis, SELLAS, Servier. The remaining authors declare no competing financial interests.

## Supporting information


**Data S1:** Supporting Information.

## Data Availability

The data that support the findings of this study are available from the corresponding author upon reasonable request.

## References

[1] C. D. DiNardo , K. W. Pratz , A. Letai , et al., “Safety and Preliminary Efficacy of Venetoclax With Decitabine or Azacitidine in Elderly Patients With Previously Untreated Acute Myeloid Leukaemia: A Non‐randomised, Open‐Label, Phase 1b Study,” Lancet Oncology 19, no. 2 (2018): 216–228. 29339097 10.1016/S1470-2045(18)30010-X

[2] C. D. DiNardo , K. Pratz , V. Pullarkat , et al., “Venetoclax Combined With Decitabine or Azacitidine in Treatment‐Naive, Elderly Patients With Acute Myeloid Leukemia,” Blood 133, no. 1 (2019): 7–17. 30361262 10.1182/blood-2018-08-868752 PMC6318429

[3] C. D. DiNardo , B. A. Jonas , V. Pullarkat , et al., “Azacitidine and Venetoclax in Previously Untreated Acute Myeloid Leukemia,” New England Journal of Medicine 383, no. 7 (2020): 617–629. 32786187 10.1056/NEJMoa2012971

[4] C. D. DiNardo , I. S. Tiong , A. Quaglieri , et al., “Molecular Patterns of Response and Treatment Failure After Frontline Venetoclax Combinations in Older Patients With AML,” Blood 135, no. 11 (2020): 791–803. 31932844 10.1182/blood.2019003988 PMC7068032

[5] N. Gangat , I. Johnson , K. McCullough , et al., “Molecular Predictors of Response to Venetoclax Plus Hypomethylating Agent in Treatment‐Naive Acute Myeloid Leukemia,” Haematologica 107, no. 10 (2022): 2501–2505. 35770533 10.3324/haematol.2022.281214 PMC9521222

[6] K. W. Pratz , B. A. Jonas , V. Pullarkat , et al., “Long‐Term Follow‐Up of VIALE‐A: Venetoclax and Azacitidine in Chemotherapy‐Ineligible Untreated Acute Myeloid Leukemia,” American Journal of Hematology 99, no. 4 (2024): 615–624. 38343151 10.1002/ajh.27246

[7] K. W. Pratz , B. A. Jonas , V. Pullarkat , et al., “Measurable Residual Disease Response and Prognosis in Treatment‐Naïve Acute Myeloid Leukemia With Venetoclax and Azacitidine,” Journal of Clinical Oncology 40, no. 8 (2022): 855–865. 34910556 10.1200/JCO.21.01546 PMC8906463

[8] A. Bataller , A. Bazinet , C. D. DiNardo , et al., “Prognostic Risk Signature in Patients With Acute Myeloid Leukemia Treated With Hypomethylating Agents and Venetoclax,” Blood Advances 8, no. 4 (2024): 927–935. 38113472 10.1182/bloodadvances.2023011757 PMC10877112

[9] N. Gangat , I. Johnson , K. McCullough , et al., “Molecular Predictors of Response to Venetoclax Plus Hypomethylating Agent in Treatment‐Naïve Acute Myeloid Leukemia,” Haematologica 107, no. 10 (2022): 2501–2505. 35770533 10.3324/haematol.2022.281214 PMC9521222

[10] N. Gangat , O. Karrar , M. Iftikhar , et al., “Venetoclax and Hypomethylating Agent Combination Therapy in Newly Diagnosed Acute Myeloid Leukemia: Genotype Signatures for Response and Survival Among 301 Consecutive Patients,” American Journal of Hematology 99, no. 2 (2024): 193–202. 38071734 10.1002/ajh.27138

[11] H. Döhner , K. W. Pratz , C. D. DiNardo , et al., “Genetic Risk Stratification and Outcomes Among Treatment‐Naive Patients With AML Treated With Venetoclax and Azacitidine,” Blood 144 (2024): 2211–2222. 39133921 10.1182/blood.2024024944 PMC11600046

[12] A. Bataller , S. Loghavi , Y. Gerstein , et al., “Characteristics and Clinical Outcomes of Patients With Myeloid Malignancies and DDX41 Variants,” American Journal of Hematology 98, no. 11 (2023): 1780–1790. 37665752 10.1002/ajh.27070 PMC11770637

[13] H. Döhner , A. H. Wei , F. R. Appelbaum , et al., “Diagnosis and Management of AML in Adults: 2022 Recommendations From an International Expert Panel on Behalf of the ELN,” Blood 140, no. 12 (2022): 1345–1377. 35797463 10.1182/blood.2022016867

[14] K. W. Pratz , P. Panayiotidis , C. Recher , et al., “Venetoclax Combinations Delay the Time to Deterioration of HRQoL in Unfit Patients With Acute Myeloid Leukemia,” Blood Cancer Journal 12, no. 4 (2022): 71. 35443742 10.1038/s41408-022-00668-8 PMC9021259

[15] D. A. Pollyea , C. D. DiNardo , M. L. Arellano , et al., “Impact of Venetoclax and Azacitidine in Treatment‐Naive Patients With Acute Myeloid Leukemia and IDH1/2 Mutations,” Clinical Cancer Research 28 (2022): 2753–2761. 35046058 10.1158/1078-0432.CCR-21-3467 PMC9365354

[16] A. Nanaa , R. He , J. M. Foran , et al., “Venetoclax Plus Hypomethylating Agents in DDX41‐Mutated Acute Myeloid Leukaemia and Myelodysplastic Syndrome: Mayo Clinic Series on 12 Patients,” British Journal of Haematology 204, no. 1 (2024): 171–176. 37710381 10.1111/bjh.19105

[17] D. A. Pollyea , K. W. Pratz , A. H. Wei , et al., “Outcomes in Patients With Poor‐Risk Cytogenetics With or Without TP53 Mutations Treated With Venetoclax and Azacitidine,” Clinical Cancer Research 28, no. 24 (2022): 5272–5279. 36007102 10.1158/1078-0432.CCR-22-1183 PMC9751752

[18] J. P. Bewersdorf , S. Shimony , R. M. Shallis , et al., “Combination Therapy With Hypomethylating Agents and Venetoclax Versus Intensive Induction Chemotherapy in IDH1‐ Or IDH2‐Mutant Newly Diagnosed Acute Myeloid Leukemia—A Multicenter Cohort Study,” American Journal of Hematology 99, no. 8 (2024): 1640–1643. 38751104 10.1002/ajh.27366

[19] J. Versluis , M. Metzner , A. Wang , et al., “Risk Stratification in Older Intensively Treated Patients With AML,” Journal of Clinical Oncology 43, no. 34 (2024): 4084–4094. 10.1200/JCO.23.02631 PMC11608593 39231389

[20] F. W. Hoff , W. Blum , Y. Huang , et al., “Beat‐AML 2024 ELN‐Refined Risk Stratification for Older Adults With Newly Diagnosed AML Given Lower‐Intensity Therapy,” Blood Advances 8 (2024): 5297–5305. 39110987 10.1182/bloodadvances.2024013685 PMC11497398

[21] G. C. Issa , J. Zarka , K. Sasaki , et al., “Predictors of Outcomes in Adults With Acute Myeloid Leukemia and KMT2A Rearrangements,” Blood Cancer Journal 11, no. 9 (2021): 162. 34588432 10.1038/s41408-021-00557-6 PMC8481264

[22] A. Tefferi , N. Gangat , A. Al‐Kali , et al., “A Dynamic 3‐Factor Survival Model for Acute Myeloid Leukemia That Accounts for Response to Induction Chemotherapy,” American Journal of Hematology 97, no. 9 (2022): 1127–1134. 35702875 10.1002/ajh.26630

[23] K. J. Norsworthy , X. Gao , C.‐W. Ko , et al., “Response Rate, Event‐Free Survival, and Overall Survival in Newly Diagnosed Acute Myeloid Leukemia: US Food and Drug Administration Trial‐Level and Patient‐Level Analyses,” Journal of Clinical Oncology 40, no. 8 (2022): 847–854. 34890212 10.1200/JCO.21.01548 PMC8906455

[24] C. S. Hourigan , L. W. Dillon , G. Gui , et al., “Pre‐MEASURE: Multicenter Evaluation of the Prognostic Significance of Measurable Residual Disease Testing Prior to Allogeneic Transplantation for Adult Patients With AML in First Remission,” Journal of Clinical Oncology 40, no. S16 (2022): 7006.

[25] M. Heuser , S. D. Freeman , G. J. Ossenkoppele , et al., “2021 Update on MRD in Acute Myeloid Leukemia: A Consensus Document From the European LeukemiaNet MRD Working Party,” Blood 138, no. 26 (2021): 2753–2767. 34724563 10.1182/blood.2021013626 PMC8718623

