# Supplementary material for: Mayo Genetic Risk Models for Newly Diagnosed Acute Myeloid Leukemia Treated With Venetoclax + Hypomethylating Agent
Source: Am J Hematol. 2024 Dec 13;100(2):260–71. doi: 10.1002/ajh.27564 (PMC11705209; doi:10.1002/ajh.27564)

**Supplemental Table 1. Predictors of** **complete response with (CR) or without (CRi) count recovery in 400 patients with newly diagnosed acute myeloid leukemia receiving venetoclax plus hypomethylating agent therapy**

| **Variables** | **CR/CRi**  **N=247 (62%)** | | | | |
| --- | --- | --- | --- | --- | --- |
|  | **Univariate**  ***P-value***  ***CR/CRi rates*** | **Mutations alone**  **Multivariate**  ***P- value***  ***(Odds Ratio)*** | **Karyotype-adjusted**  **ELN adverse**  ***P- value***  ***(Odds Ratio)*** | **Karyotype+mutations**  **Multivariate**  ***P- value***  ***(Odds Ratio)*** | **All variables**  **Multivariate**  ***P- value***  ***(Odds Ratio)*** |
| Age | 0.94 |  |  |  |  |
| Gender | 0.19  59% vs 66%  Male vs. Female |  |  |  |  |
| Secondary AML | **0.03**  52% vs. 65%  Presence vs. Absence |  | **0.01**  **(1.9)** |  | **0.03**  **(1.8)** |
| ELN 2022  adverse karyotype | **<0.01**  48% vs. 70%  Presence vs. Absence |  |  | **<0.01**  **(2.3)** | **<0.01**  **(2.3)** |
| *KMT2A* rearrangement | 0.31  43% vs 62%  Presence vs. Absence |  |  |  |  |
| *TP53* mutation | **<0.01**  46% vs. 67%  Presence vs. Absence | **<0.01**  **(3.2)** | 0.21 | **0.04**  **(1.9)** | **0.04**  **(1.9)** |
| *IDH2* mutation | **0.01**  77% vs. 60%  Presence vs. Absence | **0.08**  **(0.20)** | **0.09**  **(0.23)** | 0.22 | 0.23 |
| *IDH1* mutation | **0.09**  77% vs 61%  Presence vs. Absence | 0.26 | 0.25 | 0.37 | 0.37 |
| *NPM1* mutation | **<0.01**  84% vs. 56%  Presence vs. Absence | **0.03**  **(0.34)** | **0.01**  **(0.38)** | **0.03**  **(0.53)** | **0.04**  **(0.41)** |
| *DNMT3A* mutation | **<0.01**  77% vs.59%  Presence vs. Absence | 0.27 | **0.03**  **(0.49)** | 0.23 | 0.28 |
| *FLT3-*ITD mutation | **<0.01**  41% vs. 64%  Presence vs. Absence | **<0.01**  **(4.7)** | **<0.01**  **(3.8)** | **<0.01**  **(4.8)** | **<0.01**  **(4.8)** |
| *RUNX1* mutation | **<0.01**  49% vs. 65%  Presence vs. Absence | **<0.01**  **(2.2)** | **<0.01**  **(2.6)** | **<0.01**  **(2.4)** | **<0.01**  **(2.4)** |
| *DDX41* mutation | **<0.01**  93% vs. 61%  Presence vs. Absence | **0.05**  **(0.19)** | **0.03**  **(0.17)** | 0.09 | 0.16 |
| *KRAS* mutation | 0.49  53% vs 62%  Presence vs. Absence |  |  |  |  |
| *NRAS* mutation | 0.71  65% vs 61%  Presence vs. Absence |  |  |  |  |

Abbreviations: ELN- European LeukemiaNet

**Supplemental Table 2. Predictors of overall survival in 247 patients with newly diagnosed acute myeloid leukemia receiving venetoclax plus hypomethylating agent therapy and achieving complete response with (CR) or without (CRi) count recovery**

| **Variables** | **Overall survival**  **Transplant-censored** | | |
| --- | --- | --- | --- |
|  | **Univariate**  ***P-value***  **HR (95% CI)** | **Multivariate with all pre-treatment variables**  ***P-value***  **HR (95% CI)** | **Multivariate with genetic variables only**  ***P-value***  **HR (95% CI)** |
| Age | 0.43 |  |  |
| Gender | **<0.01**  1.8 (1.1-2.8)  Male vs female | **<0.01**  2.3 (1.4-3.7) |  |
| Secondary AML | 0.26 |  |  |
| ELN 2022 adverse karyotype | **<0.01**  2.5 (1.7-3.8)  Presence vs. Absence | **0.04**  1.8 (1.1-3.3) | **<0.01**  2.3 (1.5-3.6) |
| *KMT2A* rearrangement | **<0.01**  11.3 (2.6-48.6)  Presence vs. Absence | **<0.01**  19.5 (3.9-95.2) | **<0.01**  7.5 (1.7-32.7) |
| *TP53* mutation | **<0.01**  2.6 (1.6-4.1)  Presence vs. Absence | 0.12 | 0.14 |
| *IDH2* mutation | **<0.01**  2.8 (1.2-6.5  Absence vs. Presence | **0.03**  2.5 (1.1-5.8) | **0.01**  2.8 (1.2-6.5) |
| *IDH1* mutation | 0.08  2.0 (0.8-5.0)  Absence vs. Presence | 0.18 | 0.10 |
| *NPM1* mutation | 0.65 |  |  |
| *DNMT3A* mutation | 0.35 |  |  |
| *FLT3-*ITD mutation | 0.22 |  |  |
| *RUNX1* mutation | 0.40 |  |  |
| *DDX41* mutation | 0.31 |  |  |
| *KRAS* mutation | 0.06  2.4 (1.0-5.9)  Presence vs. Absence | **<0.01**  3.5 (1.4-8.9) | **<0.01**  3.4 (1.4-8.5) |
| *NRAS mutation* | 0.69 |  |  |
| MRD positive vs negative | 0.09 |  |  |

Abbreviations: ELN- European LeukemiaNet, MRD- measurable residual disease

**Supplemental Table 3. Predictors of overall survival in 117 patients with newly diagnosed acute myeloid leukemia receiving venetoclax plus hypomethylating agent therapy at MD Anderson Cancer Center**

| **Variables** |  | **Overall survival**  **Transplant-censored** | | |
| --- | --- | --- | --- | --- |
|  | **n(%) or median [range]** | **Univariate**  ***P-value***  **HR (95% CI)** | **Multivariate with all pre-treatment variables**  ***P-value***  **HR (95% CI)** | **Multivariate with genetic variables only**  ***P-value***  **HR (95% CI)** |
| Age | 73 [61-89] | 1.02 (0.99-1.06), p=0.23 |  |  |
| Male Gender | 71 (61) | 1.14 (0.73-1.80), p=0.57 |  |  |
| Secondary AML | 16 (14) | **1.66 (0.87-2.92), p=0.10** | **1.74 (0.91-3.10), p=0.07** |  |
| ELN 2022 adverse karyotype | 53 (45) | **2.95 (1.89-4.64), p<0.01** | 1.41 (0.74-2.73), p=0.30 | 1.36 (0.71-2.62), p=0.36 |
| *KMT2A*r | 3 (3) | 1.07 (0.18-3.40), p=0.93 |  |  |
| *TP53* mutation | 41 (35) | **4.05 (2.55-6.46), p<0.01** | **3.18 (1.68-6.24), p<0.01** | **3.19 (1.70-6.23), p<0.01** |
| *IDH2* mutation | 17 (15) | **0.36 (0.15-0.73), p=0.01** | 0.73 (0.29-1.60), p=0.47 | 0.72 (0.29-1.56), p=0.44 |
| *IDH1* mutation | 12 (10) | 0.60 (0.25-1.22), p=0.20 |  |  |
| *NPM1* mutation | 26 (22) | **0.38 (0.21-0.67), p<0.01** | 0.79 (0.38-1.60), p=0.52 | 0.78 (0.37-1.57), p=0.49 |
| *DNMT3A* mutation | 23 (20) | **0.63 (0.34-1.07), p=0.10** | 0.70 (0.37-1.22), p=0.23 | 0.70 (0.37-1.23), p=0.24 |
| *FLT3-*ITD mutation | 4 (3) | 1.69 (0.51-4.12), p=0.31 |  |  |
| *RUNX1* mutation | 14 (12) | 0.99 (0.48-1.84), p=0.98 |  |  |
| *DDX41* mutation | 3 (3) | 0.45 (0.03-2.01), p=0.42 |  |  |
| *KRAS* mutation | 6 (5) | **2.42 (0.94-5.16), p=0.04** | **4.84 (1.77-11.32), p<0.01** | **4.55 (1.67-10.59), p<0.01** |
| *NRAS mutation* | 21 (18) | 1.02 (0.55-1.76), p=0.95 |  |  |
| CR/CRi | 88 (75) | **0.18 (0.11-0.29), p<0.01** |  |  |
| MRD negative  (In responders only, 10 pts with missing data) | 42/78 (54%) | **0.43 (0.25-0.75), p<0.01** |  |  |

**Supplemental Table 4. Predictors of overall survival in 88 patients with newly diagnosed acute myeloid leukemia who achieved CR/CRi receiving venetoclax plus hypomethylating agent therapy at MD Anderson Cancer Center**

| **Variables** |  | **Overall survival**  **Transplant-censored** | | |
| --- | --- | --- | --- | --- |
|  | **n(%) or median [range]** | **Univariate**  ***P-value***  **HR (95% CI)** | **Multivariate with all pre-treatment variables**  ***P-value***  **HR (95% CI)** | **Multivariate with genetic variables only**  ***P-value***  **HR (95% CI)** |
| Age | 73 [61-89] | 1.01 (0.96-1.06), p=0.68 |  |  |
| Male Gender | 71 (61) | 1.03 (0.60-1.79), p=0.92 |  |  |
| Secondary AML | 16 (14) | **2.02 (0.92-3.95), p=0.06** | **2.81 (1.22-5.93), p<0.01** |  |
| ELN 2022 adverse karyotype | 53 (45) | **2.97 (1.74-5.12), p<0.01** | 0.98 (0.43-2.26), p=0.96 | 0.87 (0.38-1.98), p=0.73 |
| *KMT2A*r | 3 (3) | 1.65 (0.27-5.36), p=0.487 |  |  |
| *TP53* mutation | 41 (35) | **4.87 (2.75-8.60), p<0.01** | **4.04 (1.81-9.53), p<0.01** | **3.84 (1.75-8.89), p<0.01** |
| *IDH2* mutation | 17 (15) | **0.32 (0.11-0.72), p=0.01** | 0.53 (0.18-1.35), p=0.22 | 0.50 (0.16-1.26), p=0.17 |
| *IDH1* mutation | 12 (10) | **0.46 (0.14-1.14), p=0.14** | 0.43 (0.11-1.33), p=0.18 | 0.52 (0.14-1.54), p=0.28 |
| *NPM1* mutation | 26 (22) | **0.36 (0.17-0.68), p<0.01** | 0.72 (0.29-1.71), p=0.46 | 0.62 (0.27-1.42), p=0.26 |
| *DNMT3A* mutation | 23 (20) | 0.76 (0.38-1.40), p=0.41 |  |  |
| *FLT3-*ITD mutation | 4 (3) | **3.66 (0.59-12.31), p=0.08** | **13.9 (1.96-62.98), p<0.01** | **10.55 (1.51-45.98), p<0.01** |
| *RUNX1* mutation | 14 (12) | 1.39 (0.57-2.90), p=0.42 |  |  |
| *DDX41* mutation | 3 (3) | 0.65 (0.04-2.95), p=0.67 |  |  |
| *KRAS* mutation | 6 (5) | 1.31 (0.21-4.26), p=0.71 |  |  |
| *NRAS mutation* | 21 (18) | 0.83 (0.34-1.73), p=0.65 |  |  |
| MRD negative  (In responders only, 10 pts with missing data) | 42/78 (54%) | **0.44 (0.24-0.77), p<0.01** |  |  |

**Supplemental Table 5. Predictors of overall survival in 117 patients with newly diagnosed acute myeloid leukemia receiving venetoclax plus hypomethylating agent therapy at University of Chicago**

| **Variables** | **Overall survival**  **Transplant-censored** | | |
| --- | --- | --- | --- |
|  | **Univariate**  ***P-value***  **HR (95% CI)** | **Multivariate with all pre-treatment variables**  ***P-value***  **HR (95% CI)** | **Multivariate with response**  ***P-value***  **HR (95% CI)** |
| Age | 0.57 |  |  |
| Gender | 0.25 |  |  |
| Secondary AML | **0.02**  1.8 (1.1-3.0)  Presence vs. Absence | 0.04  1.7 (1.0-2.8) | 0.99 |
| ELN 2022 adverse karyotype | **<0.01**  2.4 (1.5-3.9)  Presence vs. Absence | 0.09  1.8 (0.9-3.8) | 0.23 |
| *TP53* mutation | **<0.01**  2.4 (1.5-3.7)  Presence vs. Absence | 0.41 | 0.83 |
| *IDH2* mutation | 0.49 |  |  |
| *IDH1* mutation | 0.83 |  |  |
| *NPM1* mutation | 0.41 |  |  |
| *FLT3-*ITD mutation | 0.56 |  |  |
| *K/NRAS* mutation | 0.19 |  |  |
| *RUNX1* mutation | 0.46 |  |  |
| *DDX41* mutation | 0.87 |  |  |
| CR/CRi | **<0.01**  5.9 (3.6-10) |  | **<0.01**  5.6 (3.3-9.6) |

Abbreviations: ELN- European LeukemiaNet, CR- complete remission, CRi- CR with incomplete count recovery


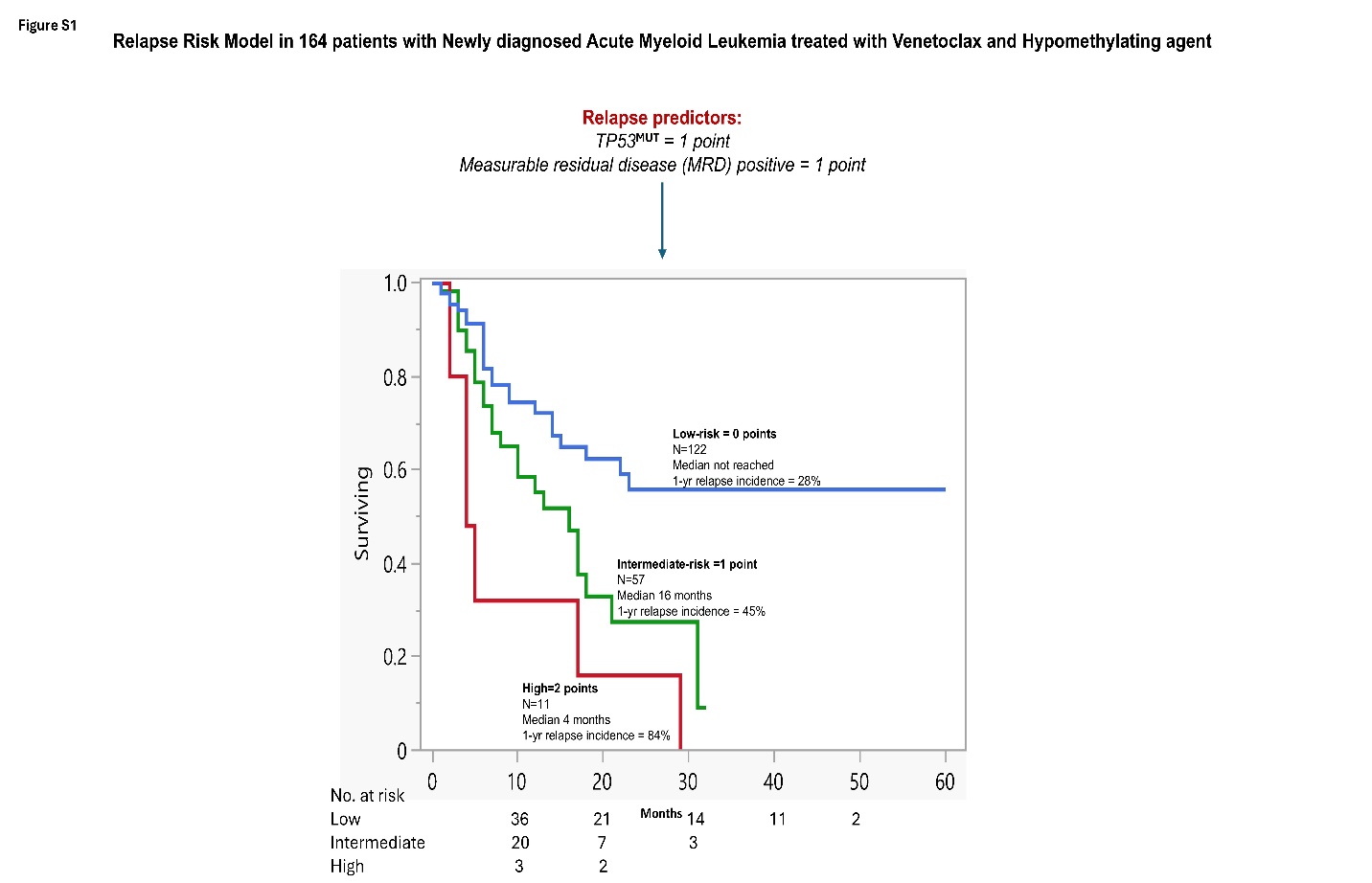


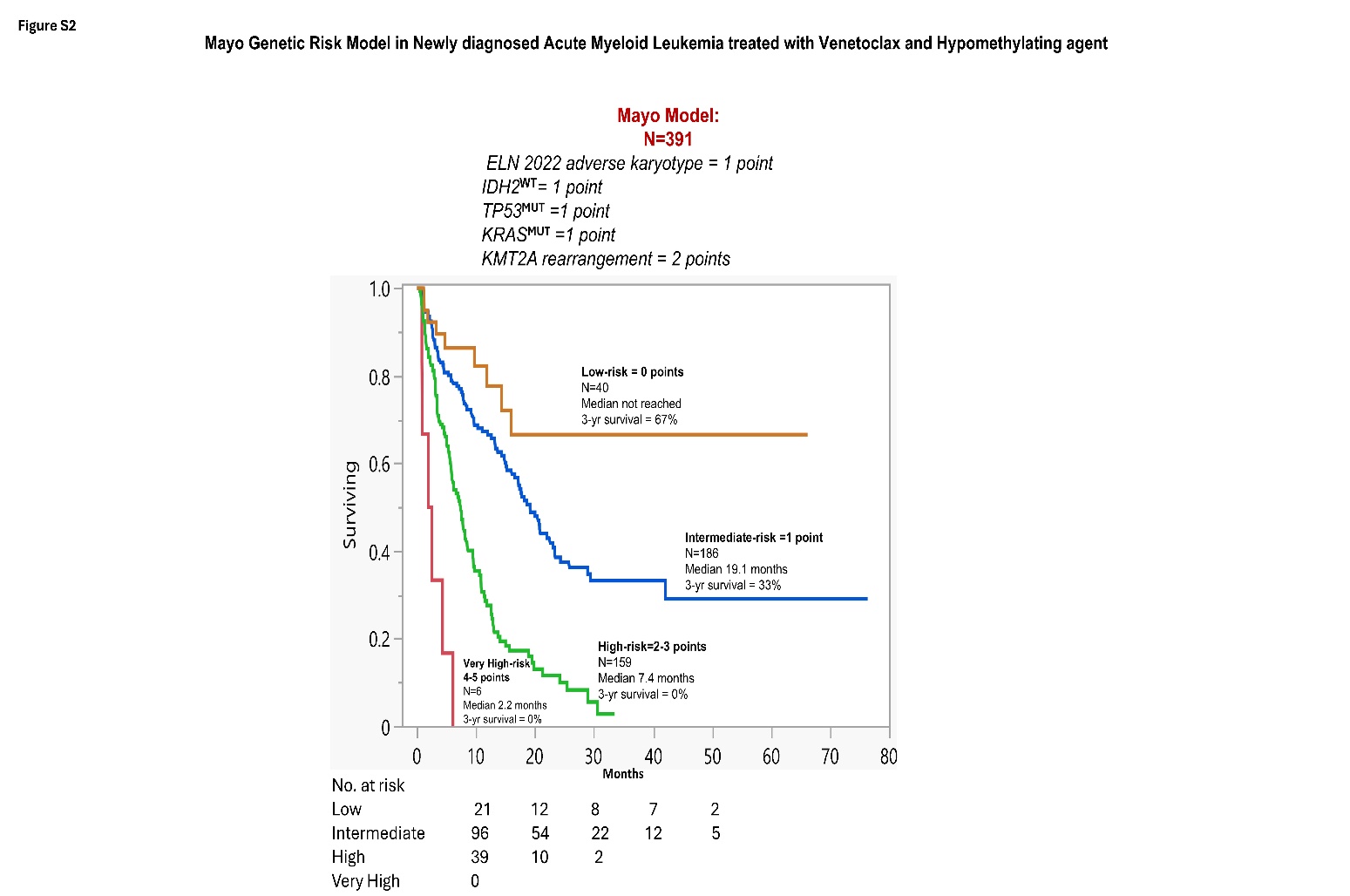


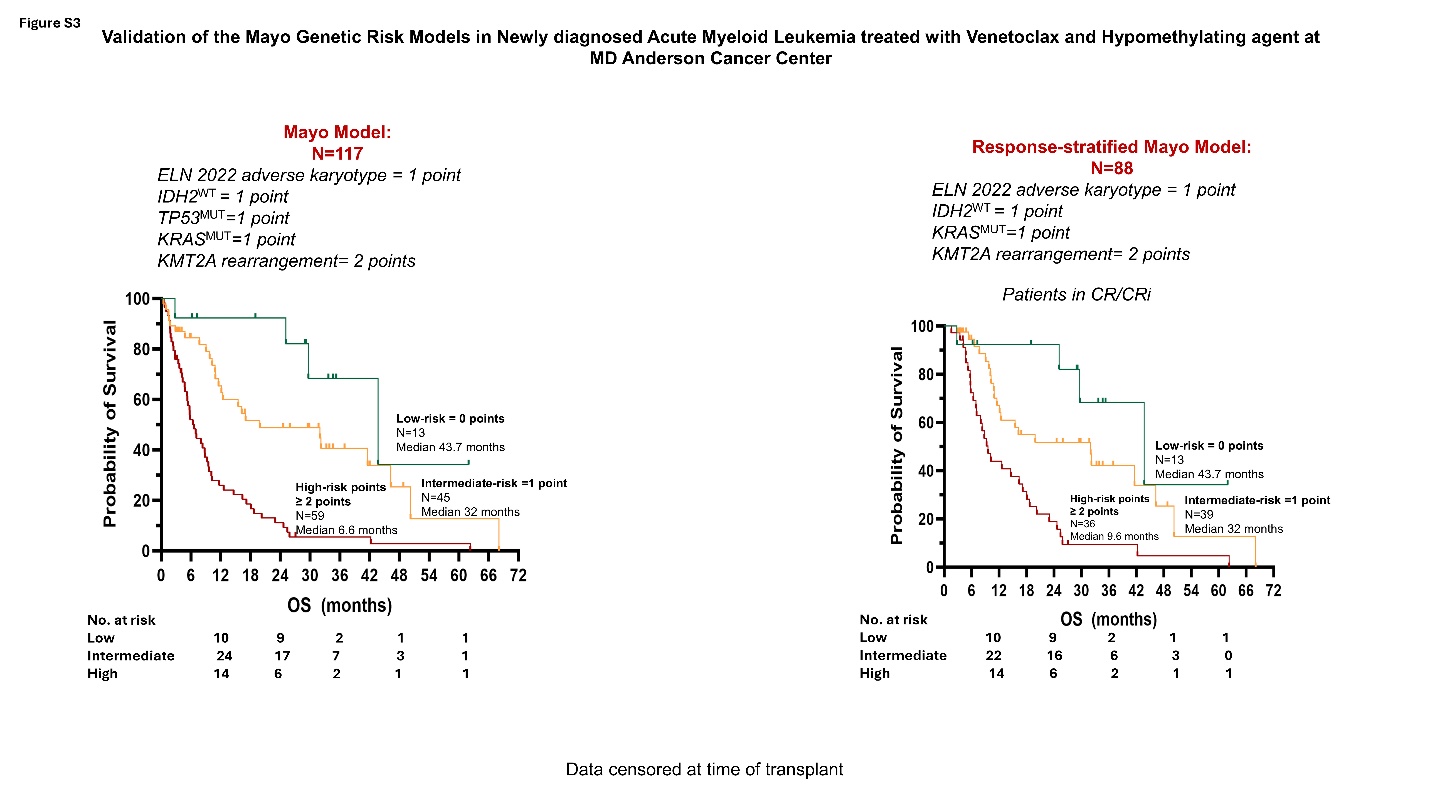

Supplement: Supplementary file 1 — Data S1: Supporting Information. [file AJH-100-260-s001.docx]
